# Transcriptional and Genomic Control of Stem Cells in Development and Cancer

**DOI:** 10.1155/2017/2513598

**Published:** 2017-06-18

**Authors:** Jinsong Zhang, Chien-Hung Gow, Sohaib Khan, Ying Liu, Chuanwei Yang

**Affiliations:** ^1^Department of Pharmacology and Physiology, Saint Louis University School of Medicine, 1402 South Grand Blvd, St. Louis, MO 63104, USA; ^2^Department of Internal Medicine, Far Eastern Memorial Hospital, New Taipei City 220, Taiwan; ^3^Department of Cell Biology, Vontz Center for Molecular Studies, University of Cincinnati College of Medicine, 3125 Eden Avenue, Cincinnati, OH 45267, USA; ^4^Center for Stem Cell and Regenerative Medicine, Mischer Neuroscience Institute, The University of Texas Health Science Center at Houston, Houston, TX 77030, USA; ^5^MD Anderson Cancer Center, Houston, TX 77030, USA

Stem cells play important roles in normal physiology, and their deregulated functions are involved in diseases such as cancer. The normal stem cells have the potential to differentiate into different cell types during development [1]. In adults, somatic stem cells are a valuable source for tissue-specific cell types to replace aged or damaged cells [2]. Cancer stem cells are cells within a tumor which possess some stem cell properties [3]. A cancer stem cell removed from its original tumor and seeded in a new organ or tissue type can form a brand new tumor in the new location, a process known as metastasis [4]. Stem cells can be regulated at both transcriptional and genomic levels. Transcription factors and their coactivators or corepressors are very important both in normal stem cell function and in cancer stem cell maintenance [5–7]. Genetic mutations resulted from replication errors or defective repair mechanisms affect genome stability and stem cell functions [8, 9]. Therapeutic targeting of stem cell regulation has the potential to alleviate disease condition and to find a cure in the fight against cancer.

This special issue contains review articles that offer new insight into the current status of the research areas. In one of the reviews, M. Wang et al. presented the common types of molecular mutations that can occur as a single mutation or as combinations of two or three mutations in cytogenetically normal acute myeloid leukemia (CN-AML). They analyzed available AML databases using bioinformatics tools at their disposal and found that mutations in stem cell regulatory factors such as FLT3, TET2, DNMT3A, and IDH1 often have an unfavorable clinical outcome and may predict relapse of leukemia in CN-AML patients. In another review, S. Zhang et al. described the cancer stem cell properties of polyploidy giant cancer cells (PGCC) and presented evidence from the literature showing that tumor budding and micropapillary pattern are recognized indicators of tumor aggressiveness in colorectal cancer. Literature review by K. M. Beach et al. discussed the role of Müller glia and Müller glial-derived stem cells in retina regeneration after injury in mammals. They summarized recent progress made in the field and emphasized that discovery of intrinsic and extrinsic factors that regulate JAK/STAT and MAPK signaling pathways take priority in future research to promote switch in the direction of regenerative responses of Müller glia in the retina after injury in mammals. In a review on neural stem cells, L. Zhang et al. summarized recent progress on the regulation of neural stem cells at both the genomic and transcriptional levels. They used bioinformatics methods to predict factors that may serve as novel therapeutic targets for functional recovery after hemorrhagic stroke. In one other review, S.-L. Cheng et al. examined 15 clinical trials using stem cells from different tissue/organ sources for the treatment of chronic obstructive pulmonary disease. Available data from three trials indicate that administration of mesenchymal stem cells (MSCs) in patients with degenerative lung diseases is well tolerated and may improve overall health and quality of life for patients.

This special issue also includes original research articles of various aspects related to the topic. L. Guo et al. observed the effect of resveratrol treatment on human umbilical cord mesenchymal stem cells (hUC-MSCs). They found that resveratrol at relatively high concentrations (15.0 and 30.0 mg/L) stimulated the hUC-MSC differentiation into neuron-like cells as evidenced by the increased expression of nestin and neuron-specific enolase (NSE) at protein and mRNA levels four hours after treatment. On the other hand, expression of glial fibrillary acidic protein (GFAP) showed no change after resveratrol treatment, indicating that differentiation of hUC-MSCs into glial cells did not occur after resveratrol stimulation. The discovery may be of therapeutic importance. One other research article by C. Yang et al. investigated the maintenance of stemness in triple-negative breast cancer (TNBC) which is enriched in cancer stem cells. They analyzed available TCGA datasets and several smaller-size datasets of breast cancer and demonstrated that cadherin family members CDH2, 4, 6, and 17 were upregulated when E-cadherin (CDH1) was lost or downregulated. Changes in the expression of CDH2/4/6/17 were associated with increased expression of several important stem cell-related transcription factors including FOXM1, MCM2, WWTR1, and Sox9. Further analysis by connectivity map search indicated that small compounds may be of therapeutic use in targeting TNBC when expression of CDHs is altered. In another research article, A. Po et al. studied the maintenance of stemness in normal cerebellar stem cells (CSC). They showed that silencing of intragenic miR-326 and its host gene *β*-arrestin1 by epigenetic modification, specifically by CpG islands methylation, of *β*-arrestin1 promoter contributes to the maintenance of stem cell properties of CSCs. They further showed that the use of demethylating agents triggered differentiation program and promoted cell growth, but inhibited cell proliferation.

In summary, enormous progress has been made in genomic and transcriptional control of stem cells in development and cancer. Reviews and original research articles presented in this special issue bring to light recent development and future challenges ahead.

*Jinsong Zhang* *Jinsong Zhang* *Chien-Hung Gow* *Chien-Hung Gow* *Sohaib Khan* *Sohaib Khan* *Ying Liu* *Ying Liu* *Chuanwei Yang* *Chuanwei Yang*
